# Efficacy of Probiotic Supplementation in Broilers Challenged with Coccidiosis: A Meta-Analysis

**DOI:** 10.1007/s12602-026-10967-2

**Published:** 2026-03-09

**Authors:** Roshan Riaz, Neslihan Ölmez, Ali Raza, Muhammad Shazaib Ramay, Benian Yılmaz, Mükremin Ölmez, Beenish Imtiaz, Hafiz Muhammad Nouman

**Affiliations:** 1 Department of Animal Nutrition and Nutritional Diseases, Faculty of Veterinary Medicine, Kafkas University, 36100 Kars, Türkiye; 2 Department of Parasitology, Faculty of Veterinary Medicine, Kafkas University, 36100 Kars, Türkiye; 3 Department of Microbiology, Faculty of Veterinary Medicine, Atatürk University, Erzurum, Türkiye; 4 Department of Animal Nutrition and Nutritional Diseases, Faculty of Veterinary Medicine, Ankara University, 06110 Ankara, Türkiye; 5 Department of Animal Nutrition and Nutritional Disease, Faculty of Veterinary Medicine, Atatürk University, Erzurum, Türkiye; 6 Department of Animal Nutrition and Nutritional Diseases, Faculty of Veterinay Medicine, Selçuk University, Konya, Türkiye

**Keywords:** Probiotics, Eimeria, Broilers, Coccidiosis, Meta-analysis, Growth performance, Lesion score, Mortality

## Abstract

Coccidiosis caused by *Eimeria* species remains a major enteric disease in broiler production, impairing growth and increasing mortality. Concerns regarding anticoccidial drug resistance have driven interest in probiotics as non-antibiotic alternatives. This meta-analysis quantitatively assessed the efficacy of probiotic supplementation in experimentally infected broiler chickens with *Eimeria* species. In accordance with the PRISMA 2020 standards, searches were conducted on September 26, 2025, in PubMed, Scopus, and Web of Science for English-language papers published between 2020 and 2025. Eligible studies included randomized controlled trials that reported quantifiable outcomes on growth performance (body weight gain [BWG], feed intake [FI], and feed conversion ratio [FCR]), or health performance (lesion score and mortality). Data were retrieved as mean ± SD or event counts and pooled using random-effects models in R (meta and metafor packages). The SYRCLE’s Risk of Bias tool was used to evaluate methodological quality. Fifteen studies comprising more than 9,000 broilers fulfilled the inclusion criteria. Probiotic supplementation markedly enhanced body weight gain (MD = 67.72 g/bird, 95% CI: 7.91 to 127.53; *p* = 0.0265, I² = 85.2%) and reduced feed conversion ratio (MD = − 0.07, 95% CI: − 0.11 to − 0.03; *p* = 0.0014, I² = 76.5%). Lesion scores significantly decreased (MD = − 0.91, 95% CI: − 1.34 to − 0.49; *p* = 0.001, I² = 95.4%), and mortality decreased by 64% (OR = 0.36, 95% CI: 0.14–0.93; *p* = 0.0399, I² = 11.1%). Probiotic supplementation had no significant impact on FI. Meta-regression demonstrated that sample size and route of probiotic administration moderated body weight gain (R² = 100%), route of administration and broiler strain explained heterogeneity in feed intake (R² = 75.39%), comparator type accounted for heterogeneity in feed conversion ratio (R² = 100%), and group size influenced lesion scores (50 birds/group: estimate = − 1.59; R² = 87.36%). Sensitivity analysis validated the robustness of the findings, and Egger’s test indicated no significant publication bias. In summary, probiotic treatment significantly improved growth performance and reduced intestinal lesion and mortality in broilers challenged with *Eimeria*. These findings support the use of probiotics as effective and sustainable alternatives to anticoccidial drugs in broilers production. However, strain-specific optimization and standardized reporting are necessary for its widespread implementation.

## Introduction

Coccidiosis is a persistent economic burden in commercial poultry production, resulting from protozoa of the genus *Eimeria*, which infects the intestinal epithelium of chickens, causing enteric lesions, malabsorption, reduced feed conversion, and mortality [1, 2]. Higher disease incidence rates are currently exacerbated globally by intensive rearing systems, high stocking densities, and environmental stressors such as heat stress. *Eimeria*-induced coccidiosis is estimated to cost up to USD 3 billion/year globally, including bird loss, reduced productivity, and treatment costs such as drugs, vaccines, and biosecurity programs [3, 4]. The increasing incidence of drug resistance in Eimeria and the impact of climate change on parasite distributions further emphasize the need for sustainable alternatives to traditional control options [5].

Chemical anticoccidials and ionophores have been the basis for prevention for decades. However, the efficacy of these products has waned due to widespread resistance and concerns over residues [6–8]. Although live and attenuated vaccines provide incomplete protection, they are currently expensive and labor-intensive to produce and may cause reversion to disease in the field [9]. In addition to this global inhibition of antibiotic growth, promotions render non-antibiotic alternatives imperative to explore. Consequently, considerable effort has been devoted to studying natural or biological substitutes for antibiotics, including probiotics, prebiotics, phytochemicals, and essential oils [10–13]. Probiotics, which are live microorganisms that exert health benefits on the host, are developed as viable and eco-friendly alternatives for poultry disease management and value addition [14].

Probiotics have multiple effects on gut physiology and the immune system. The probiotics restore microbial homeostasis, enhance epithelial tight junction integrity, and modulate both innate and adaptive immune responses [15, 16]. *Lactobacillus*-based species enhance the absorption of nutrients and repress the colonization of pathogens mainly through competitive exclusion and the production of bacteriocins. Spore-forming *Bacillus* strains stimulate butyrate production and intestinal morphometry [17]. Ahead of proprietary product development, synergistic multi-strain formulations are effective by combining the different functional features of single isolates, possessing beneficial immune-enhancing and gut-stabilizing profiles compared to single-strain products [18]. These mechanisms are correlated with better body weight gain and feed conversion efficiency, which are important productivity parameters in broiler production. However, the effects of probiotics can be strain-, dose-, and context-dependent, depending on environmental factors, management practices, and host factors [19].

Probiotic supplementation in Eimeria-challenged broilers has been investigated in multiple controlled trials, quantifying the protective efficacy of probiotics against Eimeria infections. Nooreh, Taherpour [20] and Guo, Tong [21] reported that *Lactobacillus fermentum* and *L. paracasei* significantly reduced intestinal lesion scores and oocyst shedding, increased antibody titters, and improved growth performance. Similarly, supplementation with *Bacillus subtilis* and *B. amyloliquefaciens* enhanced feed conversion and villus height, replacing antibiotics under dual coccidiosis–*Clostridium perfringens* challenge [17]. Mechanistically, these probiotics can modulate signaling pathways, including the JAK/STAT pathway, and reshape the intestinal microbiota, thereby opposing *Eimeria*-induced dysbiosis [1, 22, 23]. The findings from studies on probiotic-vaccine synergy indicate that co-administration promotes both humoral and cellular immunity and reduces lesion severity compared to vaccine-alone responses [24, 25].

Despite promising results, strain-specific differences in efficacy, performance response size, and reproducibility in experimental models remain inconsistent. In contrast, probiotics based on *Lactobacillus* have been shown to positively affect intestinal integrity, while those based on *Bacillus* have exhibited different profiles of protection depending on the challenge intensity or co-infection model being tested [22, 26]. When used in conjunction with vaccines, the addition of probiotics also failed to restore alpha diversity or growth performance in some cases significantly [25]. Such inconsistencies highlight the potential of systematic evidence synthesis to reconcile heterogeneity and elucidate patterns of benefit. In addition, environmental and managerial factors, such as stocking density, hygiene, and litter moisture, regulate infection pressure and probiotic efficacy; however, such context-dependent effects are poorly quantified [19].

To address these gaps, a meta-analysis was conducted to assess the effect of probiotic supplementation on growth performance in broiler chickens following experimental challenge with *Eimeria* using a quantitative approach. This analysis collected data from multiple randomized controlled trials. It incorporated evidence on important production and disease-related endpoints, such as body weight gain, feed intake, feed conversion ratio, lesion score, and mortality, while simultaneously exploring the effects of study-level moderators, such as sample size, sample size per group, and comparator type. This study aimed to deliver a systematic, statistical, and integrative synthesis that clarifies the potential of probiotics against coccidiosis, identifies uncertainties that remain, and outlines future avenues in the application of microbiome-based strategies for sustainable broiler production.

## Methodology

### Search Strategy

**Fig. 1:**
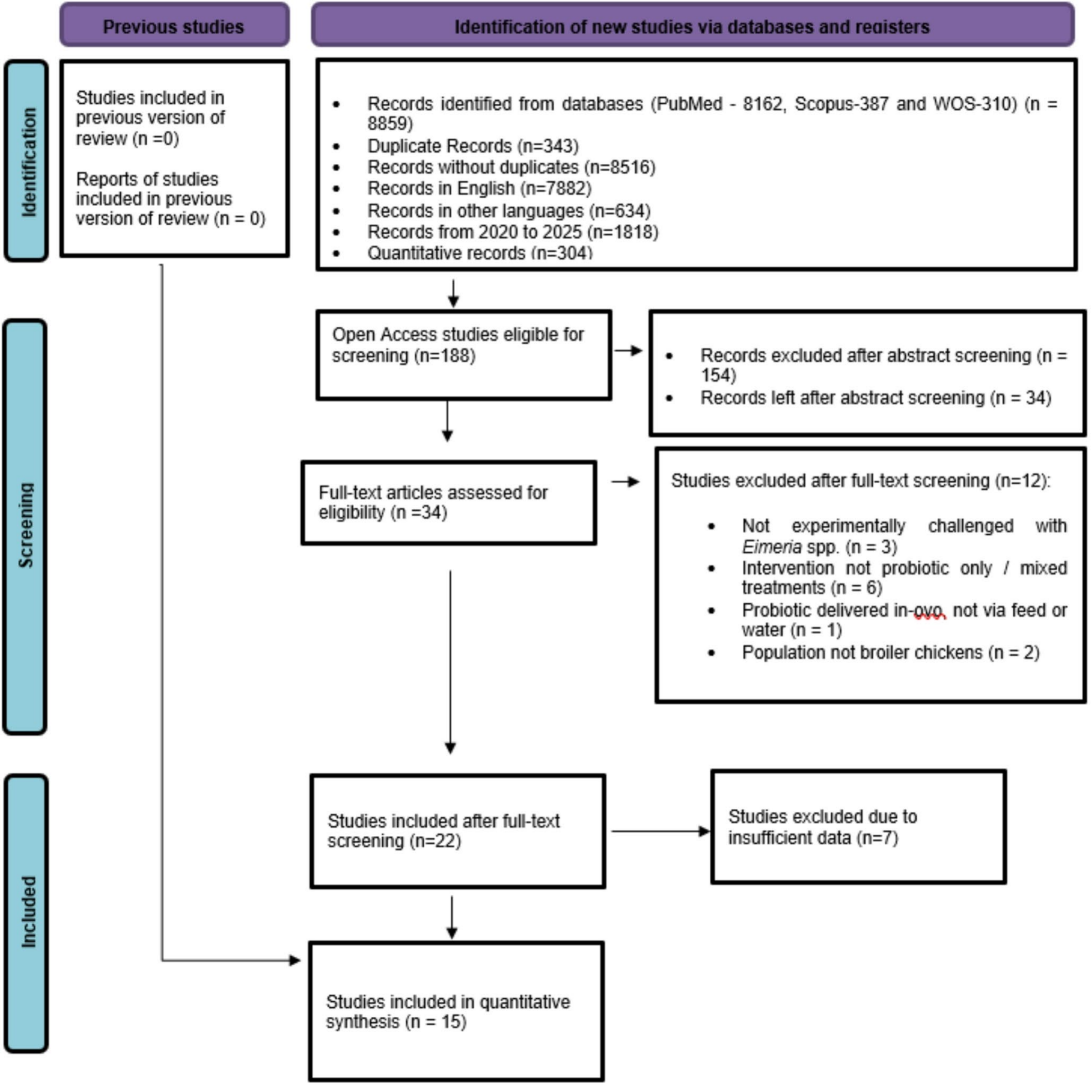
PRISMA flow diagram showing the identification, screening, and inclusion of studies

A PRISMA 2020 (Fig. 1) compliant systematic literature search was performed on September 26, 2025 to screen studies assessing probiotic supplementation in broiler chickens challenged with *Eimeria* species under experimental conditions [27]. Controlled vocabulary and keyword combinations to capture all relevant studies were used for systematic searches in three major databases: PubMed, Scopus, and Web of Science. The final search strings were as follows:

#### PubMed

(probiotics [MeSH Terms] OR probiotic [Title/Abstract] OR *Lactobacillus* [Title/Abstract] OR *Bacillus* [Title/Abstract] OR *Saccharomyces* [Title/Abstract]) AND (broiler [Title/Abstract] OR broilers [Title/Abstract] OR chicken [Title/Abstract] OR poultry [Title/Abstract]) AND (coccidiosis [MeSH Terms] OR coccidiosis [Title/Abstract] OR *Eimeria* [Title/Abstract].

#### WOS

TS= (probiotic* OR *Lactobacillus* OR *Bacillus* OR *Saccharomyces*) AND TS= (broiler* OR chicken* OR poultry) AND TS= (coccidiosis OR *Eimeria*).

#### SCOPUS

TITLE-ABS-KEY (probiotic* OR *Lactobacillus* OR *Bacillus* OR *Saccharomyces*) AND TITLE-ABS-KEY (broiler* OR chicken* OR poultry) AND TITLE-ABS-KEY (coccidiosis OR *Eimeria*).

The search was limited to peer-reviewed and English-language articles and included records published from 2020 to 2025. After duplicate removal, all records were imported to the reference manager (Endnote) to keep them organized for subsequent screenings [28].

### Pico Statement and Research Question

This meta-analysis was conducted following the PICO framework to define the research question and eligibility criteria.

#### Population (P)

Broiler chickens (*Gallus gallus domesticus*) experimentally challenged with *Eimeria* species.

#### Intervention (I)

Probiotic supplementation administered through feed or water, including strains of *Lactobacillus*, *Bacillus*, and *Saccharomyces*.

#### Comparator (C)

Non-supplemented control groups, including negative controls, standard diets, or infected control groups without probiotic supplementation.

#### Outcomes (O)

Growth performance outcomes (body weight gain, feed intake, and feed conversion ratio) and health performance outcomes (intestinal lesion score and mortality).

Based on this framework, the primary research question was: Does probiotic supplementation improve growth performance and health outcomes in broiler chickens experimentally challenged with *Eimeria* species compared with non-supplemented controls?

### Inclusion Criteria

Eligible studies were required to satisfy several criteria about methodology and reporting. The population of concern consisted of broiler chickens *(Gallus gallus domesticus)* that had been experimentally challenged with a species of *Eimeria*, with detailed characterization reports of the infecting species, challenge, dose, and timing. The intervention of interest in this meta-analysis was probiotic supplementation only, whether delivered through feed or water. Probiotics, including strains of *Lactobacillus*,* Bacillus*,* and Saccharomyces*, were included in the study. Trials using multi-strain products or mixed agents were considered only if the effects of the probiotics could be clearly distinguished from those of the other interventions. The comparator consisted of a non-supplemented placebo, standard diet, or control group to estimate the relative treatment effects. Studies were included in the meta-analysis if statistical data on at least one of the predetermined primary outcomes classified into growth performance and health performance were available. Growth performance was evaluated using body weight gain (BWG), feed intake (FI), and feed conversion ratio (FCR), and health performance was assessed based on mortality (%) and lesion scores. For every outcome, statistical data such as mean ± standard deviation (SD) or standard error (SE) and sample size (n) were required for both the treatment and control groups. Studies that presented 95% confidence intervals were also included. The number of events or proportions was considered in relation to mortality. Only studies with an in vivo randomized controlled trial or controlled observational design that clearly reported the units of analysis (pen or bird) were included. Finally, no other types of studies, except peer-reviewed journal articles, were included to ensure that the methodology was sound and reproducible.

### Exclusion Criteria

Studies that did not align with the eligibility criteria in any key aspect were excluded. This exclusion criterion primarily focused on studies conducted in poultry types other than broilers, such as layers, turkeys, or quails, as well as studies that lacked a clear *Eimeria* challenge. The exclusion criteria included interventions other than probiotics, such as pre-biotics, syn-biotics, postbiotics, antibiotics, phytogenic additives, enzymes, organic acids, and herbal extracts, as well as other interventions. To minimize the risk of confounding, studies with combination treatments were excluded if the effects of probiotics could not be clearly distinguished from those of other co-interventions. Studies from which it was impossible to extract the quantitative data needed to calculate the effect size, such as studies reporting only p-values, graphical data without numeric values, or medians with interquartile ranges, were excluded from the analysis. Studies that reported purely immune, oxidative, or histological data without growth or health performance outcomes were excluded. The final analysis also excluded non-primary research, including reviews, meta-analyses, conference abstracts, theses, and case reports. To maintain the internal validity and comparability of the studies, the predefined criteria were strictly applied.

### Data Extraction and Organization

Data extraction was performed independently and systematically using a standardized form designed for this meta-analysis. This was performed in two sequential stages. In stage one, study characteristics were extracted, such as study identifier (author and year of publication), study design, broiler strain and age, total sample size (N), and experimental design characteristics (Table 1). Data were collected on the *Eimeria* challenge administered, including species, challenge dose, and age at infection. The details of the probiotic intervention were thoroughly extracted, including microbial species or strain, dosage (CFU/g), route of administration (feed or water), and duration of supplementation. Comparator types, such as negative control, positive control, and antibiotic-treated groups, were also extracted. Stage two involved extracting the quantitative outcome data necessary for the meta-analysis. The data was extracted as means ± standard deviations for both treatment and control groups for continuous outcomes, including BWG, FI, FCR, and lesion score. For dichotomous outcomes (mortality), the number of events (deaths) and the total number of birds at risk in the treatment and control groups were extracted to calculate odds ratios. All data extracted from the studies were organized into a standard data matrix with the following columns: study ID, study design, study design, strain, sample size (total and per group), type of challenge, probiotic, comparator, duration, outcome values, effect type, and measurement unit. Mean difference (MD) was used for continuous outcomes when measurement units and scales were identical across studies. Body weight gain was consistently reported in g/bird, and feed conversion ratio in g/g. Lesion scores were assessed using the same ordinal scale (0–4) across all included studies; therefore, MD was considered appropriate and clinically interpretable. Odds ratios (OR) were used for dichotomous outcomes (mortality). Formula for MD and OR are given below:

**Mean Difference (MD).**

For continuous outcomes measured on the same scale:

$$\mathrm{MD}={\stackrel{\prime }{X}}_{\mathrm{treatment}}-{\stackrel{\prime }{X}}_{\mathrm{control}}$$

${\stackrel{\prime }{X}}_{\mathrm{treatment}}$= mean outcome in the probiotic group.

${\stackrel{\prime }{X}}_{\mathrm{control}}$= mean outcome in the control group.

**Odds Ratio (OR)**.

For dichotomous outcomes (mortality):

$$\mathrm{OR}=\frac{a/b}{c/d}=\frac{a\times\;d}{b\;\times\;c}$$

- $a$= number of events in the treatment group.
- $b$= number of non-events in the treatment group.
- $c$= number of events in the control group.
- $d$= number of non-events in the control group.

### Risk of Bias Assessment

SYRCLE’s Risk of Bias (ROB) tool, an adaptation of the Cochrane Collaboration’s instrument specific for animal intervention studies, was used to assess the methodological quality of the studies and potential sources of bias in all included studies [29]. The tool evaluates ten domains: sequence generation, baseline comparability of groups, allocation concealment, random housing, blinding of caregivers and investigators, random outcome assessments, blinding of outcome assessors, completeness of outcome data, selective outcome reporting, and other potential biases. The domains were classified as low, unclear, or high risk of bias based on the methodological details reported for each study. A risk-of-bias assessment was performed independently by two reviewers, with disagreements resolved through discussion to achieve consensus. SYRCLE’s Risk of Bias tool was applied with full domain-level reporting for all included studies. In addition, a conservative worst-domain approach was used to derive an overall study-level risk of bias classification for descriptive and sensitivity purposes only, whereby a study was considered at high risk if at least one domain was rated as high risk. This overall judgment did not replace domain-specific assessments but was used to facilitate transparent interpretation of study quality in quantitative synthesis. Detailed domain-level results are presented in Sect.  3.3 and Table 2.

### Statistical Analysis

All statistical analyses were performed in the R software environment (version 4.4.2; R Foundation for Statistical Computing, Vienna, Austria) using the *meta* [30] and *metafor* [31] packages for quantitative synthesis [32]. The studies were categorized into two primary outcomes: growth performance and health performance. Random-effects models were applied for all meta-analyses to account for between-study heterogeneity arising from differences in experimental design, sample size, group size, and comparator type [33]. For continuous outcomes measured on identical scales across studies, effect sizes were expressed as mean differences (MDs) with 95% confidence intervals, while odds ratios (ORs) were used for dichotomous outcomes (mortality). Statistical heterogeneity was assessed using the I² and τ² statistics. Potential publication bias was evaluated using visual inspection of funnel plots and Egger’s regression test. When evidence of asymmetry was detected, the trim-and-fill method was applied as a sensitivity analysis [34]. The robustness of pooled estimates was further examined using leave-one-out sensitivity analyses [35]. To explore sources of heterogeneity, predefined study-level moderators, including comparator type (infected untreated controls, positive/antibiotic controls, and other disease-specific controls) as a study-level moderator to account for heterogeneity arising from differing experimental control designs and was evaluated using meta-regression, along with total sample size, group size, route of administration, and broiler strain [36]. Statistical significance was defined as *p* < 0.05 for all analyses.

## Results

### Studies Selection

The systematic search across PubMed, Scopus, and Web of Science identified 8,859 records. After excluding 343 duplicates, 8,516 articles were screened. After excluding 634 non-English records, 7882 studies in English were included. Further restricting the search by publication year (2020–2025) yielded 1,818 articles, of which 304 were randomized controlled or observational studies. Non-original publications, including reviews, conference papers, and theses (*n* = 1,514), were excluded. A total of 188 studies were eligible for abstract screening, of which 34 were retained for full-text evaluation. Nineteen of these were excluded for predefined reasons: lack of experimental *Eimeria* challenge 3 [37–39]; non-probiotic or mixed interventions 6 [40–45]; in-ovo probiotic administration 1 [46]; non-broiler populations 2 [47, 48]; and insufficient quantitative data for meta-analysis 7 [49–55]. Finally, 15 studies met al.l eligibility criteria and were included in the quantitative meta-analysis. The entire selection process, including identification, screening, eligibility, and inclusion phases, is summarized in the PRISMA 2020 flow diagram (Fig. 1).

### Study Characteristics

A total of 15 randomized or controlled trials, available in the literature from 2020 to 2025, with more than 9000 broilers from a variety of commercial strains, including Ross 308, Cobb 500, Arbour Acres, and Yellow-feathered lines, were considered for the meta-analysis. Most studies employed mixed *Eimeria* challenges, primarily consisting of *E. tenella*, *E. acervulina*, and *E. maxima*, and tested probiotics based on *Lactobacillus*,* Bacillus*,* Bifidobacterium*, or *Saccharomyces* species added to feed or water. Controls comprised uninfected/infected untreated controls, as well as occasionally anticoccidials or antibiotics, such as amprolium, lasalocid, and diclazuril. The key characteristics of the studies are presented in Table (1).

**Table 1:** Characteristics of Studies Included in the Meta-Analysis Evaluating Probiotic Supplementation in Broilers Challenged with *Eimeria* Spp

Study ID (Author, Year)	Population & Design	Eimeria Challenge (species, dose)	Intervention (Probiotic: strain/species, dosage, duration)	Comparator (negative control, positive control, antibiotics if any)
Aida, Yamada [56]	Exp 1: Ross 308, *N* = 60 (6 × 10); Exp 2: Ross 308, *N* = 90 (3 × 10 triplicate); 0–31 d & 0–49 d	Oral at d 21: *E. tenella* (5 × 10³) + *E. acervulina* (1 × 10⁵) oocysts/bird	*Weizmannia coagulans* SANK70258, 5 × 10⁴–1 × 10⁶ CFU/g feed; 0.005–0.1% inclusion; 0–31 d / 0–49 d	Negative: no additive; Positive: lasalocid-A Na 75 ppm; AM group as antibiotic
Chalalai, Promsut [57]	Ross broilers, *N* = 90 (6 × 15; 3 replicates), 1–28 d	*E. tenella* 2 × 10⁴ oocysts/bird by gavage d 15	Multispecies (Vetafarm Probiotic: *Lactobacillus acidophilus*,* L. bulgaricus*,* L. plantarum*,* L. rhamnosus*,* Bifidobacterium bifidum*,* E. faecium*,* S. thermophilus*); 1 mg/mL water; 10–28 d	Neg: uninfected/untreated; Pos: infected/untreated; Amprolium 20 mg/kg; therapeutic and prophylactic arms
Chen, Lv [58]	Cobb 500 males, *N* = 180 (3 groups, 6 × 10); 0–28 d	d 14: mixed *Eimeria* (4 × 10⁴ total oocysts) + *C. perfringens* 4 × 10⁸ CFU/mL d 19–21	*Lactobacillus plantarum* HW1 (CGMCC 26160); 4 × 10⁶ CFU/g feed, 0–28 d	Neg: non-infected basal diet; Pos: infected no probiotic; no antibiotic reported
de Souza, Vecchi [59]	Exp 1: Cobb 500 males *N* = 750 (5 × 6 × 25); Exp 2: *N* = 400 (4 × 5 × 20); 1–42 d	Exp 1: vaccine (×10 dose) + *C. perfringens* 3.1 × 10⁹ CFU/mL; Exp 2: recycled litter (~ 75,000 oocysts/g)	Zymospore^®^ (*B. subtilis* BS-009, BS-020, BS-024); 0.2–0.4 kg/ton feed; d 1–42	Neg: basal diet; Pos: AGP (Flavomycin 0.025 kg/t); BMD ± challenge (0.5 kg/t)
Li, Zheng [60]	Ross 308; *N* = 336 (4 × 6 × 14); 35 d	d 9: vaccine 30× dose (*E. tenella*,* E. necatrix*,* E. maxima*,* E. acervulina*) + *C. perfringens* 13–18 d	*L. fermentum* or *L. paracasei* 1 × 10⁹ CFU/g feed; d 1–35	Neg: CTR (no infection); Pos: CCP (challenged no probiotic); no antibiotic
Nooreh, Taherpour [61]	Ross 308 males; two experiments (*N* = 350 each); 1–42 d (floor pens) and 1–24 d (cages)	Mixed *Eimeria* (~ 50,000 *E. acervulina*, 10,000 *E. maxima*, 5,000 *E. tenella*); oral gavage d 10–14	PrimaLac (*L. casei*,* L. acidophilus*,* B. bifidum*,* S. faecium*,* A. oryzae*); 1 g/kg feed; d 1–end	Neg: uninfected; Pos: infected; Salinomycin 60 mg/kg; vit E + C + Se comparators
Ogwiji, Jatau [62]	Cobb 500 mixed-sex; *N* = 90 (6 × 3 × 5); 35 d	*E. tenella* 2 × 10⁴ oocysts d 21	*Saccharomyces cerevisiae* (Antox); 1 mL/L drinking water; d 0–35	Neg: non-infected; Pos: infected; Amprolium HCl 0.012%; prebiotic/synbiotic arms
Osho, Bolek [63]	Ross 708 males; *N* = 120 (3 × 8 × 5); 0–13 dpi	Mixed *Eimeria* via Coccivac B52^®^ (15× dose, 1 mL gavage d 11)	Multi-strain Bacillus (MicroLife^®^ Prime: *B. subtilis*,* B. licheniformis*,* B. amyloliquefaciens*,* B. coagulans*); 5 × 10⁵ CFU/g feed; 7 d pre-challenge to 13 dpi	Neg: uninfected basal; Pos: infected basal; no antibiotics reported
Poudel, Tabler [64]	Ross 708 males; *N* = 1,248 (96 pens, 13/pen); 0–41 d	COCCIVAC^®^-B52 20× dose d 14 (*E. acervulina*,* E. maxima*,* E. mivati*,* E. tenella*)	*Bacillus subtilis* PB6 (CLOSTAT^®^); ~1 × 10⁸ CFU/kg feed; 0–41 d	Diet without *B. subtilis* (± challenge); no antibiotic; outcomes: BW, FCR, mortality
Sun, Liu [65]	Yellow-feathered broilers; *N* = 600 (5 × 6 × 20); 1–80 d	d 24: 10× anticoccidial vaccine + *C. perfringens* 3 × 10⁸ CFU/mL d 28–30	*Bacillus subtilis* QST713 (≥ 1 × 10¹⁰ CFU/g); 100 mg/kg feed; d 1–80	Neg: basal diet; Pos: CIC.p and CP-only challenges; no antibiotic
Van der Klein, Arora [66]	Ross 308 males; *N* = 900 (3 × 12 × 25); 0–42 d	*E. tenella*,* E. maxima*,* E. acervulina* 4,000 oocysts/bird d 15 + APEC d 7 + *C. perfringens* d 18–20	*L. acidophilus* AG01 + *B. animalis* AG02 (1 × 10⁸ CFU/bird/day via water); d 0–42	Neg: non-challenged; Pos: challenged; no antibiotic reported
van der Klein, Bernardeau [68]	Ross 308 and Cobb 500 males; *N* = 1,440/trial (3 × 12 × 40); 0–42 d	*E. tenella*,* E. maxima*,* E. acervulina*; 10× dose B52 vaccine d 14 (as NE model)	*L. acidophilus* AG01 + *B. animalis* AG02; 1 × 10⁸–10⁹ CFU/bird/day via waterline; d 0–42	Neg: non-challenged; Pos: challenged; no antibiotic reported
Wang, Lv [67]	Arbor Acres males; *N* = 500 (5 × 10 × 10); 0–42 d	d 14 gavage 1 mL saline with 4 × 10⁴ oocysts (*E. tenella*,* E. necatrix*,* E. maxima*,* E. acervulina*)	*L. plantarum* P8; 1 × 10⁷ or 10⁸ CFU/g feed; 0–42 d	NC: non-infected; IC: infected; Pos: diclazuril 0.2 g/kg; no antibiotic
Wang, Xu [68]	Lingnan Yellow broilers; *N* = 360 (3 × 6 × 20); 0–63 d	d 15: 20× vaccine (*E. tenella*,* E. necatrix*,* E. maxima*,* E. acervulina*) + *C. perfringens* 2 × 10⁸ CFU/mL d 18–21	*B. subtilis* DSM29784; 1 × 10⁹ CFU/kg feed; d 22–63	Neg: unchallenged; Pos: vaccine + CP challenge; no antibiotic
Zhang, Yang [69]	Three-yellow chickens; *N* = 288 (12 × 3 × 8); 0–28 d	*E. maxima* 30,000 oocysts d 14 + *C. perfringens* 1 × 10⁸ CFU/mL d 18–20	*B. subtilis* (1 g/kg feed); d 1–28; alone and with plant extracts	Neg: uninfected; Pos: infected; Drug control: enramycin 10 mg/kg; no other antibiotics

### Risk of Bias Assessment

**Fig. 2:**
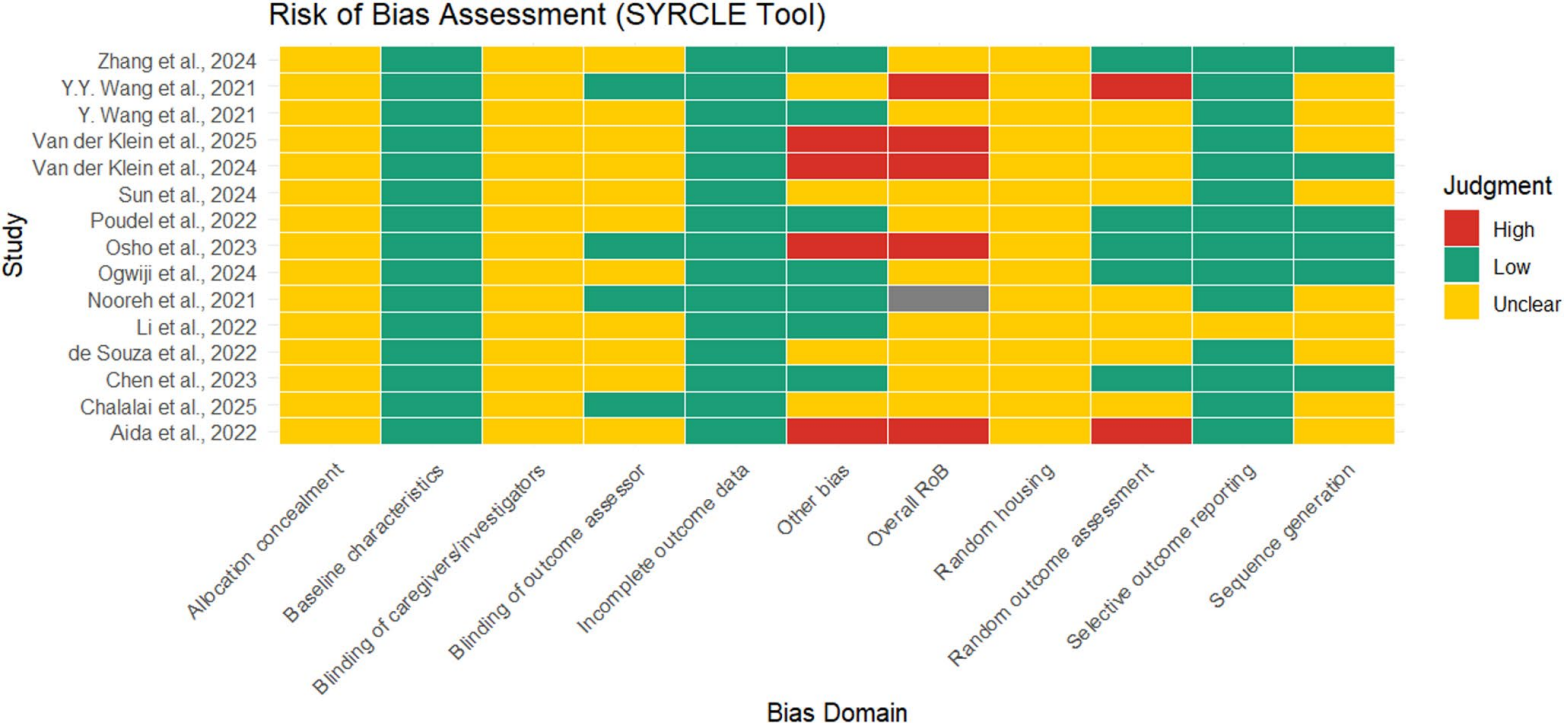
Risk of Bias assessment of the included studies using the SYRCLE tool across all methodological domains

The risk of bias was assessed using the SYRCLE Risk of Bias tool among the 15 included studies. Overall, baseline findings, outcome reporting, and data completeness across studies were rated as low risk. However, the approach to random sequence generation, allocation concealment, and blinding was often inadequately described, resulting in an unclear rating. Fewer studies were at high risk of performance and detection bias due to the lack of blinding. The distribution of risk of bias judgments across domains and studies is presented in Fig. 2.

### Growth Performance

#### Body Weight Gain (BWG)

**Fig. 3:**
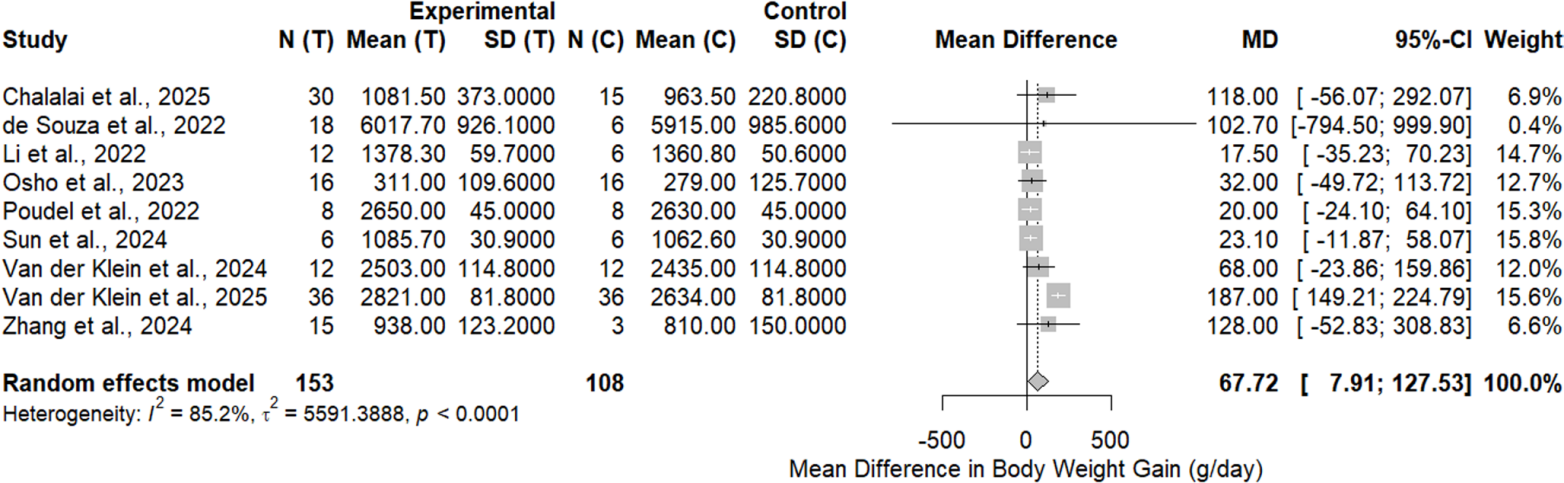
Forest plot of the effect of probiotic supplementation on body weight gain in broiler chickens challenged with *Eimeria spp*

The pooled meta-analysis of nine eligible studies revealed that probiotic supplementation significantly improved body weight gain in broiler chickens experimentally challenged with *Eimeria* species. Using the random-effects model, the mean difference was 67.72 g/bird (95% CI: 7.91 to 127.53; *p* = 0.0265). Substantial between-study heterogeneity was observed (I² = 85.2%, τ² = 5591.39, *p* < 0.0001). Meta-regression analyses identified sample size and route of probiotic administration as significant sources of between-study heterogeneity in body weight gain. Both moderators explained all observed heterogeneity (R² = 100%), with no residual between-study variability. The forest plot of individual and pooled effect sizes is presented in Fig. 3.

**Fig. 4:**
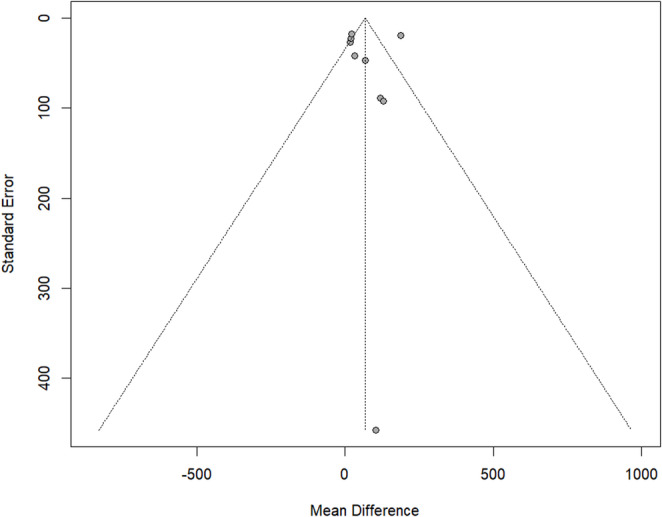
Funnel plot assessing potential publication bias in studies reporting body weight gain outcomes

Sensitivity analysis demonstrated the robustness of the findings. Sequential omission of individual studies did not substantially alter the pooled estimates, which consistently remained significant and in the same direction. The largest shift was observed when excluding van der Klein, Bernardeau [70], which reduced the pooled effect to 27.78 g/bird (95% CI: 5.56, 50.01; *p* = 0.0143) and eliminated heterogeneity (I² = 0%). However, no single study disproportionately influenced the overall results. Funnel plot visualization suggested symmetry, and Egger’s regression test confirmed the absence of small-study effects (intercept = 69.75, SE = 50.64, *p* = 0.981). The funnel plot illustrating publication bias assessment is shown in Fig. 4.

#### Feed Intake (FI)

**Fig. 5:**
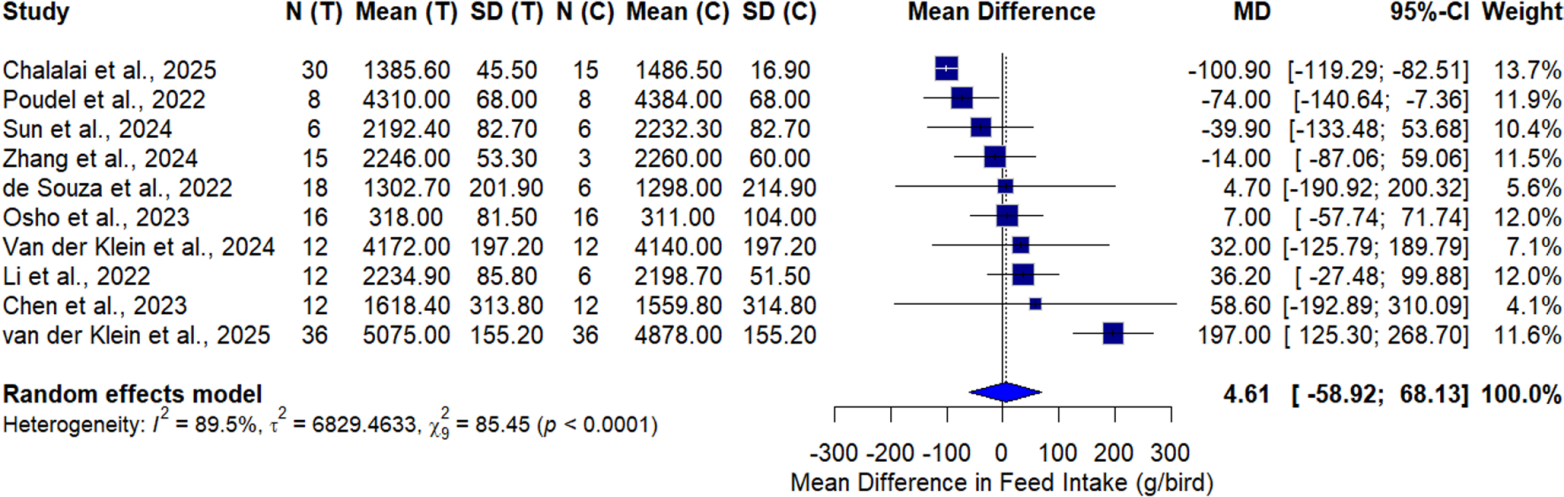
Forest plot of the effect of probiotic supplementation on feed intake in broiler chickens challenged with Eimeria spp

The meta-analysis of ten studies evaluating the effect of probiotic supplementation on feed intake in broiler chickens challenged with *Eimeria* species indicated no significant overall effect. Using a random-effects model, the pooled mean difference was 4.61 g/bird (95% CI: − 58.92, 68.13; *p* = 0.873), with substantial heterogeneity detected across studies (I² = 89.5%, τ² = 6829.46, *p* < 0.0001). Individual study estimates varied considerably, ranging from reductions in feed intake (–100.9 g/bird in Chalalai, Promsut [57]; − 74.0 g/bird in Poudel, Tabler [64] to increases (+ 197.0 g/bird in van der Klein, Bernardeau [70]. A combined meta-regression incorporating route of probiotic administration and broiler strain as moderators explained a substantial proportion of between-study heterogeneity in feed intake outcomes (R² = 75.39%). The moderator model was statistically significant (QM = 20.53, *p* = 0.0045), with residual heterogeneity reduced to a moderate level (I² = 44.79%) and no significant unexplained heterogeneity remaining (QE = 3.03, *p* = 0.2198), indicating that variability across studies was largely attributable to differences in administration route and broiler strain. The forest plot of the pooled analysis is shown in Fig. 5.

**Fig. 6:**
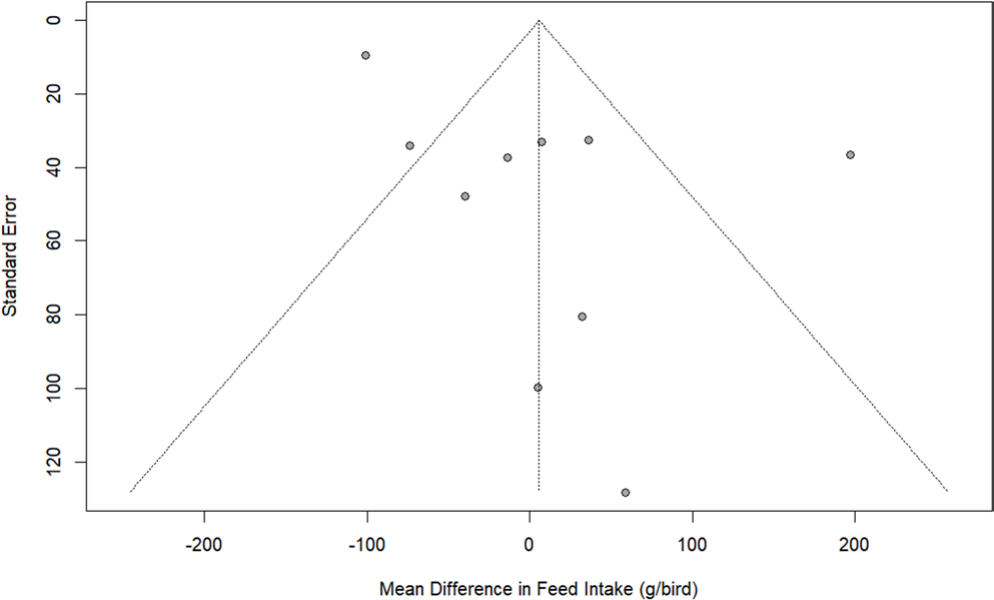
Funnel plot for feed intake (FI) studies assessing potential publication bias

**Fig. 7:**
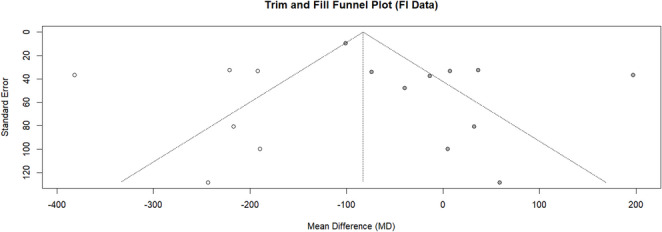
Trim-and-fill funnel plot showing adjusted estimates for feed intake outcomes

Sensitivity analysis using the leave-one-out approach confirmed the robustness of the findings. Sequential omission of individual studies did not materially change the direction or significance of the pooled effect, which consistently remained non-significant. The greatest influence was observed when excluding van der Klein, Bernardeau [70], which reduced heterogeneity (I² = 75.1%) and shifted the pooled estimate to − 27.87 g/bird (95% CI: − 69.91, 14.18; *p* = 0.165). Publication bias assessment suggested potential asymmetry. Egger’s regression test indicated small-study effects (t = 2.48, df = 8, *p* = 0.038), and trim-and-fill analysis imputed six missing studies. After adjustment, the pooled effect shifted to − 82.78 g/bird (95% CI: − 161.78, − 3.77; *p* = 0.041), indicating a significant reduction in feed intake when accounting for possible unpublished data. The funnel plot for publication bias assessment is presented in Fig. 6 and trim and fill funnel plot is presented in Fig. 7.

#### Feed to Conversion Ratio (FCR)

**Fig. 8:**
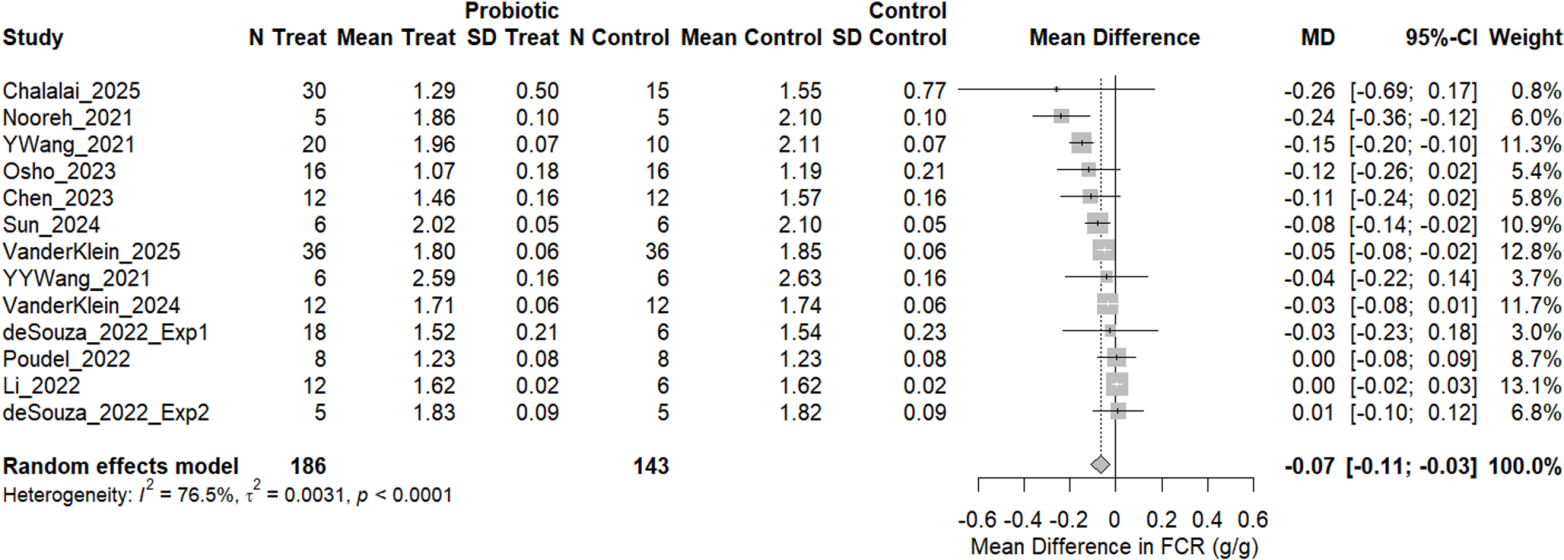
Forest plot of the effect of probiotic supplementation on feed conversion ratio (FCR) in broilers

A total of 12 studies with broilers (186 probiotic-treated and 143 controls) were included in the meta-analysis. The pooled estimate under a random-effects model demonstrated a significant reduction in FCR with probiotic supplementation compared with control groups (MD = − 0.07; 95% CI: − 0.11, − 0.03; *p* = 0.0014). Considerable heterogeneity was detected (I² = 76.5%; τ² = 0.0031; *Q* = 50.98, *p* < 0.0001). Meta-regression with comparator type as a moderator accounted for all residual heterogeneity (R² = 100%) and revealed significant effects for studies using infected controls (IC) and positive controls (POS) as comparators (QM = 50.61, *p* < 0.0001). The summary of study-level and pooled estimates is presented in the forest plot **(**Fig. 8**)**.

**Fig. 9:**
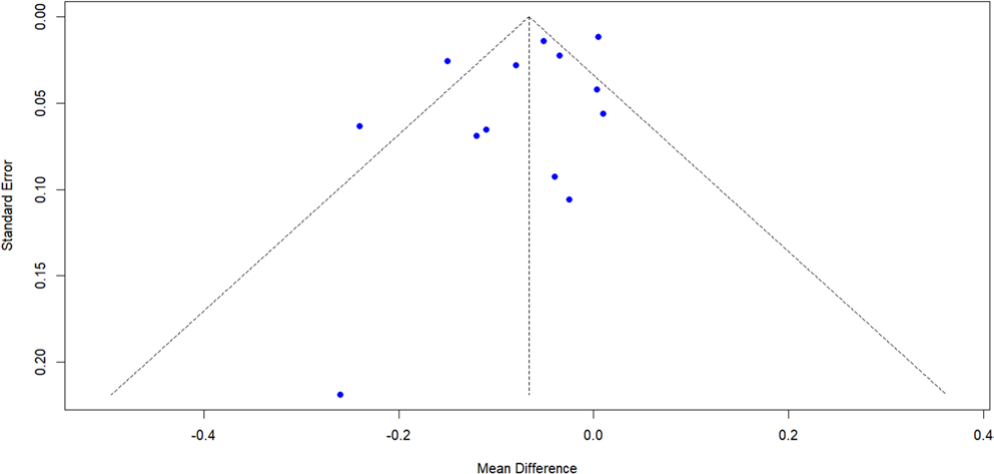
Funnel plot for feed conversion ratio (FCR) studies assessing potential publication bias

Leave-one-out sensitivity analysis showed consistent results, with pooled mean differences ranging from − 0.08 to − 0.05 across iterations, indicating that no single study disproportionately influenced the overall effect. Heterogeneity values remained stable within the range of 61.0% to 78.4%. Egger’s test for funnel plot asymmetry was not significant (intercept = − 0.01; 95% CI: − 0.055, 0.034; *p* = 0.132), suggesting no evidence of small-study effects. The funnel plot displayed symmetrical distribution of studies around the pooled effect estimate, further indicating the absence of substantial publication bias **(**Fig. 9**)**.

### Health Performance

#### Lesion Score

**Fig. 10:**
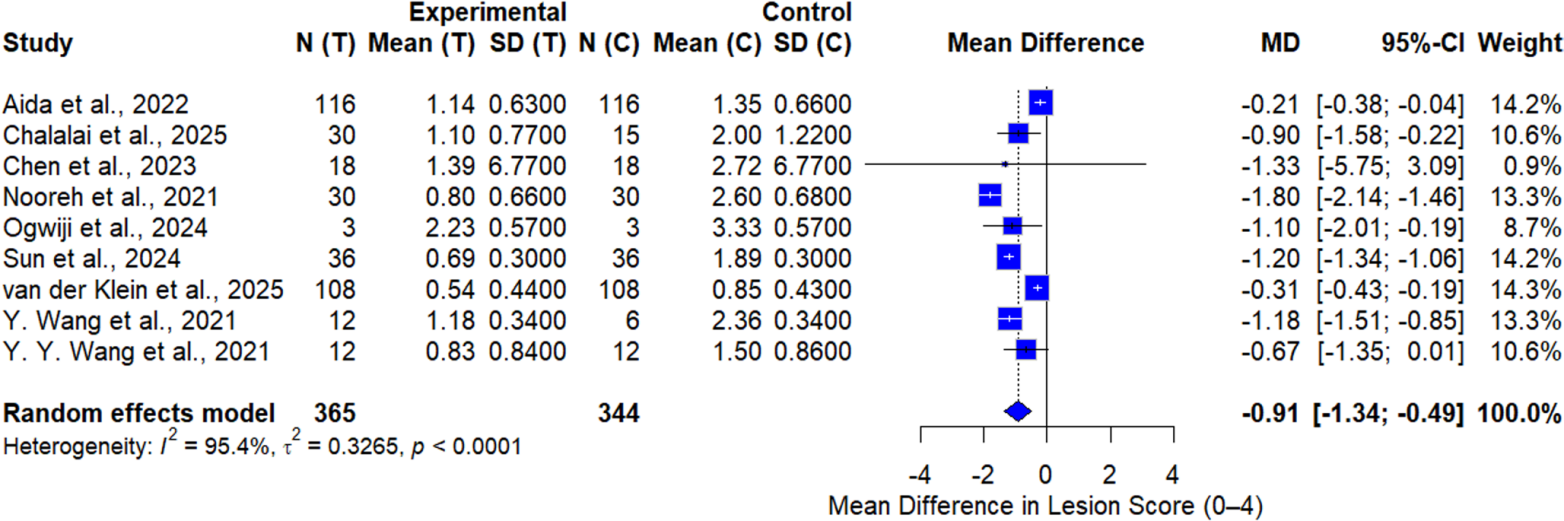
Forest plot of lesion score outcomes across nine studies (probiotics vs. control)

The pooled analysis of nine studies (365 treatment and 344 control birds) showed a significant reduction in lesion scores with probiotic supplementation, with a mean difference of − 0.91 [95% CI: −1.34, − 0.49]; *p* = 0.001 **(**Fig. 10**)**. Considerable heterogeneity was observed across the included studies (I² = 95.4%, τ² = 0.3265, Q = 175.68, df = 8, *p* < 0.0001). Meta-regression using group size as a moderator indicated that sample size explained 87.36% of the heterogeneity, reducing residual I² to 27.8%.

**Fig. 11:**
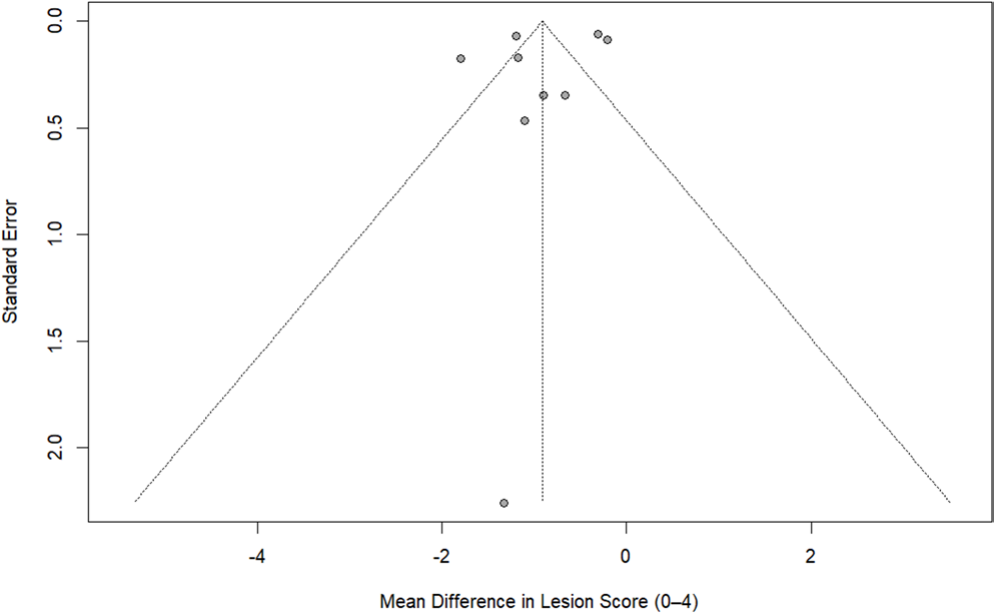
Funnel plot assessing publication bias for lesion score studies

Sensitivity analysis demonstrated stability of the pooled effect estimate, with lesion score reductions ranging from − 0.76 to − 1.03 across leave-one-out iterations, and heterogeneity estimates remained consistently high (I² = 92.8%–96.0%). Egger’s regression test for funnel plot asymmetry did not indicate significant small-study effects (t = − 0.85, df = 7, *p* = 0.4252). The funnel plot **(**Fig. 11**)** showed a generally symmetrical distribution of studies around the pooled effect.

#### Mortality

**Fig. 12:**
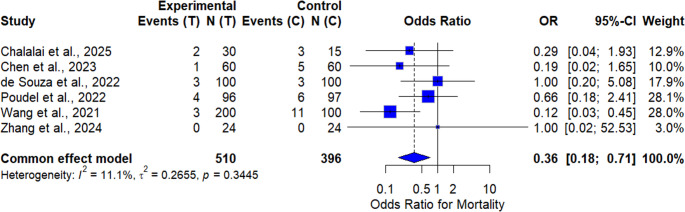
Forest plot of the effect of probiotic supplementation on broiler mortality compared with challenged controls (odds ratio of 95% CI)

The pooled analysis of six studies (510 probiotics vs. 396 control) reported 42 mortality events. The common-effects model indicated a significant reduction in mortality with probiotics (OR = 0.36, 95% CI: 0.18–0.71, *p* = 0.0033). Between-study heterogeneity was low (I² = 11.1%, τ² = 0.2655, Q = 5.62, *p* = 0.3445), suggesting stable results across trials. The forest plot is shown on Fig. 12.

**Fig. 13:**
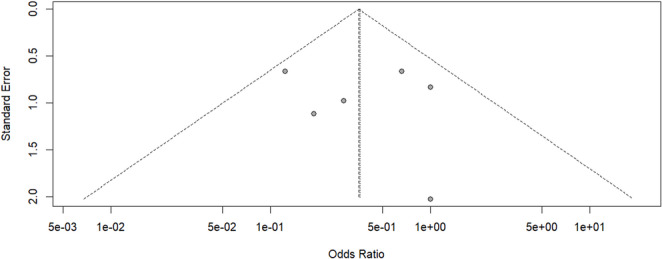
Funnel plot assessing potential publication bias in studies reporting mortality outcomes following probiotic supplementation

Leave-one-out analysis confirmed that the pooled estimate remained stable across exclusions, with ORs ranging from 0.28 [0.12–0.63] (after omitting Poudel, Tabler [64]) to 0.54 [0.24–1.21] (after omitting Wang, Lv [67]). No single trial substantially altered the direction or significance of the effect. The funnel plot appeared symmetric **(**Fig. 13**)**, and Egger’s test indicated no evidence of small-study effects (t = 0.32, df = 4, *p* = 0.7626). These findings support the robustness and reliability of the mortality effect estimates.

## Discussion

The current meta-analysis aimed to pool available quantitative data from 15 controlled trials to estimate the effectiveness of probiotic supplementation in broilers challenged with *Eimeria* species. The results showed that probiotics significantly increased BWG and FCR and decreased intestinal lesion scores and mortality rates. The overall effect on feed intake (FI) was inconsistent. Together, these data indicate that probiotics efficiently prevent the adverse effects associated with coccidiosis, improving both growth and health performance under infectious challenges. The strength of these conclusions was confirmed by the low publication bias across most outcomes and the consistent direction of the effect in the sensitivity analyses.

The increase in BWG and FCR observed in the present study is consistent with the increasing experimental evidence. Arczewska-Włosek, Świątkiewicz [71] demonstrated that multi-strain probiotic (Protexin^®^) supplementation mitigated the reduction in growth rate induced by anticoccidial vaccination to a level like that observed in unvaccinated controls for FCR and BWG. Similarly, Mohsin, Zhang [72] also found that *Lactobacillus plantarum* (1 × 10⁸ CFU/g) increased feed efficiency and decreased oocyst shedding in *E. tenella*-infected broilers. In contrast, Wang, Lv [73] reported similar findings for *L. plantarum* P8, with improvements in average daily weight and a reduction in mortality. The findings from each study align with the overall average of + 67.72 g/bird for BWG and − 0.07 for FCR derived from the current meta-analysis. This suggests that the effect is more pronounced on nutrient utilization and intestinal recovery following Eimeria-induced damage, rather than being a direct consequence of feed intake, as no difference in FI was observed.

The decrease in intestinal lesion scores found here (mean difference = − 0.91) demonstrates the protective effect of probiotics on gut integrity. Similar results were reported by Nooreh, Taherpour [20], who found that Primalac supplementation decreased duodenal and cecal lesion scores, which is comparable to the effect of salinomycin antibiotic. Ciszewski, Jarosz [40] also noted a notable reduction in lesion scores and mortality in broilers feed a probiotic + clinoptilolite combination. However, Sun, Liu [65] observed that Bacillus subtilis QST713 enhanced feed efficiency and alleviated gut damage in yellow-feathered broilers co-infected with *coccidia* and *Clostridium perfringens*. Importantly, these beneficial effects arise through strain- and genus-specific mechanisms: Lactobacillus- and Enterococcus-based probiotics primarily exert their effects via competitive exclusion of pathogens, lactic acid and short-chain fatty acid production, and modulation of mucosal immunity, whereas *Bacillus* species additionally contribute through enzyme secretion, spore-mediated resilience, and enhanced epithelial repair. These consistent findings underscore the contribution of probiotics to the restoration and maintenance of intestinal homeostasis through multiple mechanisms, including the inhibition of pathogenic species, the production of lactic acid and short-chain fatty acids, the elevation of mucosal immunity, and the stimulation of epithelial repair. Collectively, these actions account for the uniform attenuation of lesion severity reported across studies and the strong pooled response observed in this study.

The meta-analysis also reported a marked decrease in the mortality rate (OR = 0.36), indicating a 64% reduction in the odds of dying from probiotic-supplemented broilers compared with infected controls. Wang, Lv [73] and Ciszewski, Jarosz [40] both reported similar declines in mortality, while Sun, Liu [65] reported better survival among *B. subtilis*-fed broilers under mixed coccidial and bacterial challenge conditions. Although Shah, Al Hakeem [74] concluded that synbiotic supplementation had no influence on mortality during *Eimeria–Clostridium* co-infection, the observed difference in outcome is likely not contradictory and more likely the result of variation in probiotic composition and the severity of infection. The general decrease in mortality, supported by several independent studies, indicates that probiotics not only support nutrient utilization but also enhance birds’ ability to cope with diseases. Ogbuewu, Mabelebele [75] agreed with this finding, highlighting that *Bacillus*-based probiotics might be good non-antibiotic alternatives for improving productivity under infectious stress.

Considerable variety among studies (I² > 70%) had to be anticipated owing to differences in probiotic composition, challenge model, and trial conditions. Moderator analyses found that the total sample size, group sample size, comparator type, route of administration and broiler strain were statistically significant sources of heterogeneity. Trials with higher numbers of participants tended to yield smaller effect sizes, and their results likely correspond to more accurate and less random estimates. In contrast, studies that used infected or positive controls showed larger performance than those with uninfected groups as control populations. Meta - regression of lesion scores revealed that group size accounted for 87% of the heterogeneity, suggesting a trial scale effect on the reproducibility and reliability of the effects. Although other factors, such as probiotic species, and dosage were tested, their moderator effect was non-significant. These findings highlight the importance of methodological harmonization and transparent reporting in probiotic trials.

Despite the overall coherence of the results, some limitations must be mentioned. The few eligible studies reporting outcomes, especially mortality and lesion scores, may restrict statistical precision. Furthermore, missing methodological details, including randomization, allocation concealment, and blinding, resulted in numerous “unclear” judgments in the SYRCLE risk-of-bias tool, potentially leading to modest overestimates of accurate effect sizes. Small-study effects for feed intake outcomes lend evidence to the fact that publication bias could not be entirely ruled out. Finally, most of the included trials were short-term (28–42 days) experiments performed under more experimental than commercial field conditions, further restricting the transferability of the results. This should be considered when extrapolating previous findings to commercial poultry production. The results of this meta-analysis, supported by recent experimental evidence, indicate that the addition of probiotics significantly improves growth performance, feed conversion ratio, and survival, while mitigating the damage caused by *Eimeria spp*. in the intestines of broilers. The consistent response to multiple genera of probiotics, including *Lactobacillus*,* Bacillus*,* Enterococcus*, and *Saccharomyces*, also endorses their real application as a sustainable alternative or feed additive for anticoccidial drugs. Nonetheless, larger, long-term, and well-documented trials are needed to develop more informed recommendations regarding ideal dosage, strains, and application modalities under commercial conditions.

## Conclusion

This meta-analysis provides quantitative evidence that probiotic supplementation effectively improves growth performance and health in broilers undergoing experimental *Eimeria spp*. Infection. The pooled results showed a significant increase in weight gain and feed conversion ratio, as well as a significantly lower lesion score and mortality rate, indicating that probiotics can effectively combat coccidiosis. These positive effects were consistent at the genus level (*Lactobacillus*,* Bacillus*,* Enterococcus*, and *Saccharomyces*) and across diverse experimental designs, indicating the potentially wide-ranging generality of probiotic effectiveness in poultry production systems. Although inconsistent, most responses observed with feed intake suggest that probiotics primarily improve nutrient uptake and gut integrity rather than acting as direct appetite stimulants. The moderator analyses (meta – regression) distinguished sample size, group size, route of administration, broiler strain and comparator type as sources of heterogeneity, demonstrating the role of trial design in influencing the measured outcomes. The conclusions are reliable, given the high explained heterogeneity, robust pooled effects, and lack of significant publication bias. Despite this, future research must still elucidate some gaps, such as the optimal dose and duration of supplementation necessary for efficacy per *Eimeria* strain, as well as the impact of performance and robustness in the presence of conventional anticoccidial programs. Large-scale, well-designed trials under commercial conditions are necessary to sustain the long-term effectiveness and sustainability of probiotics as alternatives to antibiotics. In conclusion, this meta-analysis provides strong evidence to recommend the strategic implementation of probiotics in coccidiosis management programs as a sustainable measure to improve the growth, health, and welfare of broilers.

## Data Availability

This meta-analysis exclusively utilized previously published data and analysis data is available within the manuscript.

## References

[1] Madlala T, Okpeku M, Adeleke MA (2021) Understanding the interactions between Eimeria infection and gut microbiota, towards the control of chicken coccidiosis: a review. Parasite 28:48. 10.1051/parasite/202104734076575 10.1051/parasite/2021047PMC8171251

[2] Ahmad R, Yu Y-H, Hua K-F, Chen W-J, Zaborski D, Dybus A, Hsiao FS-H, Cheng Y-H (2023) Management and control of coccidiosis in poultry—A review. Anim Biosci 37:1–15. 10.5713/ab.23.018937641827 10.5713/ab.23.0189PMC10766461

[3] Franzo VS, Vidotti AP, Piedade AR, de Oliveira LP, Parazi YA, Mascarenhas LJS, Vulcani VAS, Ferreira JMN (2025) Advancements in technology for diagnosing and controlling coccidiosis in poultry: A review of the eimeria genus. ARACÊ 7:3533–3543. 10.56238/arev7n1-210

[4] Dinasarki D, Tenrisanna V, Amrawaty AA (2024) Broiler product quality: The global scientific research landscape and implications for marketing performance. Int J Agric Biosci 13:306–312. 10.47278/journal.ijab/2024.122

[5] Gao Y, Sun P, Hu D, Tang X, Zhang S, Shi F, Yan X, Yan W, Shi T, Wang S (2024) Advancements in understanding chicken coccidiosis: from Eimeria biology to innovative control strategies. One Health Adv 2:6. 10.1186/s44280-024-00039-x

[6] Mansoor M, Jamil M, Khan A, ul Haq R, Anwar F (2017) Control of Avian Coccidiosis: Present and Future Strategies for Natural Alternatives of Therapeutics. Biol Sci-PJSIR 60:49–62

[7] Hayajneh F, Abdelqader A, Zakaria H, Abuajamieh M, Araj S (2024) Drug resistance and coccidiosis affects immunity, performance, blood micronutrients, and intestinal integrity in broiler chickens. Int J Vet Sci 13:34–41. 10.47278/journal.ijvs/2023.054

[8] Yardimci M, Yağcilar Ç, Polat C (2025) Acute and Chronic Toxicity of the Coccidiostat Amprolium to Daphnia magna and Its Implications for Aquatic Contamination from Livestock Waste. Kafkas Univ Vet Fak Derg 31:689–696. 10.9775/kvfd.2025.34670

[9] Ghattas M, Dwivedi G, Lavertu M, Alameh M-G (2021) Vaccine technologies and platforms for infectious diseases: current progress, challenges, and opportunities. Vaccines 9:1490. 10.3390/vaccines912149034960236 10.3390/vaccines9121490PMC8708925

[10] Hussain K, Abbas A, Rehman A, Waqas MU, Ahmad B, Mughal MAS, Abbas RZ, Zaman MA, Khan JA, Raza MA (2024) Evaluating Linum usitatissimum seeds extract as potential alternative biochemical and therapeutic agent against induced coccidiosis in broiler chicken. Kafkas Univ Vet Fak Derg 30:803–808

[11] Abbas A, Hussain K, Aleem MT, Sugiharto S, Song H, Mares MM (2025) Immunomodulatory Potential of Sugar Beet (Beta vulgaris) Against Coccidiosis in Broiler Chickens. Kafkas Univ Vet Fak Derg 31:419–424. 10.9775/kvfd.2025.33760

[12] Sultana MA, Habib MA, Amin MN, Sabuz SH, Salma U, Begum MD, Haque MA (2025) Effects of yogurt on growth performance, carcass traits, lipid profile and fecal microbial load of broiler chickens. Int J Agric Biosci 14:50–58. 10.47278/journal.ijab/2024.196

[13] Hailat A, Abdelqader A, Gharaibeh M (2024) Efficacy of phyto-genic products to control field coccidiosis in broiler chickens. Int J Vet Sci 13:266–272. 10.47278/journal.ijvs/2023.099

[14] Alagawany M, Abd El-Hack ME, Farag MR, Sachan S, Karthik K, Dhama K (2018) The use of probiotics as eco-friendly alternatives for antibiotics in poultry nutrition. Environ Sci Pollut Res 25:10611–10618. 10.1007/s11356-018-1687-x10.1007/s11356-018-1687-x29532377

[15] Phupaboon S, Hashim FJ, Punyauppa-Path S, Phesatcha B, Kanpipit N, Kongtongdee P, Phumkhachorn P, Rattanachaikunsopon P (2024) Supplementation of microencapsulated fish-derived probiotic lactic acid bacteria to enhance antioxidant activity in animal feed. Int J Agric Biosci 13:250–258. 10.47278/journal.ijab/2024.110

[16] Mayasari N, Ismiraj MR, Kumalasari C, Adriani L (2025) Effects of Different Culture Media of Lactic Acid Bacteria on Performance, Carcass Yield, Blood Parameters, and Natural Antibodies in Broiler Chickens. Kafkas Univ Vet Fak Derg 31:467–476. 10.9775/kvfd.2025.33784

[17] Berto R, Junior NR, Andrade TS, Kaufmann C, Sartor H, Costa APGC, Comin GN, Toniazzo G, de Queiroz Teixeira V, Naranjo VD (2024) Bacillus-based probiotics on broiler chicken performance under coccidiosis and Clostridium perfringens challenge. S Afr J Anim Sci 54:509–523

[18] Kwoji ID, Aiyegoro OA, Okpeku M, Adeleke MA (2021) Multi-strain probiotics: synergy among isolates enhances biological activities. Biology 10:322. 10.3390/biology1004032233924344 10.3390/biology10040322PMC8070017

[19] Acharya K, Acharya N (2017) Alternatives to fight against coccidiosis: A review. Nep Vet J. 10.3126/nvj.v34i0.22918. 34:152 – 67

[20] Nooreh Z, Taherpour K, Ghasemi HA, Akbari Gharaei M, Shirzadi H (2021) Protective and immunostimulatory effects of in-feed preparations of an anticoccidial, a probiotic, a vitamin-selenium complex, and Ferulago angulata extract in broiler chickens infected with Eimeria species. BMC Vet Res 17:307. 10.1186/s12917-021-03005-634526018 10.1186/s12917-021-03005-6PMC8442408

[21] Guo S, Tong W, Qi Y, Jiang M, Li P, Zhang Z, Hu Q, Song Z, Ding B (2023) Effects of dietary limosilactobacillus fermentum and lacticaseibacillus paracasei supplementation on the intestinal stem cell proliferation, immunity, and ileal microbiota of broiler chickens challenged by coccidia and Clostridium perfringens. Animals 13:3864. 10.3390/ani1324386438136901 10.3390/ani13243864PMC10740854

[22] Memon FU, Yang Y, Zhang G, Leghari IH, Lv F, Wang Y, Laghari F, Khushk FA, Si H (2022) Chicken gut microbiota responses to dietary Bacillus subtilis probiotic in the presence and absence of Eimeria infection. Microorganisms 10:1548. 10.3390/microorganisms1008154836013966 10.3390/microorganisms10081548PMC9412415

[23] Sapsuha Y, Sundari S, Nur A (2025) Using Lactobacillus plantarum and Tomi-Tomi Fruit Extract Synbiotics as a Natural Supplement in Broiler Chickens: Impact on Blood Profile, Gut Microbiota, and Performance. Int J Vet Sci 14:1130–1136. 10.47278/journal.ijvs/2025.069

[24] Ritzi MM, Abdelrahman W, Van-Heerden K, Mohnl M, Barrett NW, Dalloul RA (2016) Combination of probiotics and coccidiosis vaccine enhances protection against an Eimeria challenge. Vet Res 47:111. 10.1186/s13567-016-0397-y27825377 10.1186/s13567-016-0397-yPMC5101694

[25] Nahed A, El-Naggar K, El-Kasrawy NI, Elblehi SS, Albadrani GM, Al-Ghadi MQ, Abdel-Daim MM (2024) The anticoccidial effects of probiotics and prebiotics on the live coccidia vaccine and the subsequent influence on poultry performance post-challenge with mixed Eimeria species. Poult Sci 103:104283. 10.1016/j.psj.2024.10428339305616 10.1016/j.psj.2024.104283PMC11437767

[26] Menconi A, Sokale AO, Mendoza SM, Whelan R, Doranalli K (2020) Effect of Bacillus subtilis DSM 32315 under different necrotic enteritis models in broiler chickens: a meta-analysis of five independent research trials. Avian Dis 64:379–385. 10.1637/aviandiseases-D-19-0011633205174 10.1637/aviandiseases-D-19-00116

[27] Page MJ, McKenzie JE, Bossuyt PM, Boutron I, Hoffmann TC, Mulrow CD, Shamseer L, Tetzlaff JM, Akl EA, Brennan SE (2021) The PRISMA 2020 statement: an updated guideline for reporting systematic reviews. BMJ 372. 10.1136/bmj.n7110.1136/bmj.n71PMC800592433782057

[28] Bramer WM, Giustini D, De Jonge GB, Holland L, Bekhuis T (2016) De-duplication of database search results for systematic reviews in EndNote. J Med Libr Assoc: JMLA 104:240. 10.3163/1536-5050.104.3.01427366130 10.3163/1536-5050.104.3.014PMC4915647

[29] Hooijmans CR, Rovers MM, De Vries RB, Leenaars M, Ritskes-Hoitinga M, Langendam MW (2014) SYRCLE’s risk of bias tool for animal studies. BMC Med Res Methodol 14:43. 10.1186/1471-2288-14-4324667063 10.1186/1471-2288-14-43PMC4230647

[30] Balduzzi S, Rücker G, Schwarzer G (2019) How to perform a meta-analysis with R: a practical tutorial. BMJ Ment Health 22:153–160 10.1136/ebmental-2019-300117 PMC10231495 31563865

[31] Viechtbauer W (2010) Conducting meta-analyses in R with the metafor package. J Stat Softw 36:1–48. 10.18637/jss.v036.i03

[32] Team RC (2021) R: A language and environment for statistical computing. R foundation for statistical computing, Vienna, Austria

[33] DerSimonian R, Laird N (1986) Meta-analysis in clinical trials. Control Clin Trials 7:177–1883802833 10.1016/0197-2456(86)90046-2

[34] Egger M, Smith GD, Schneider M, Minder C (1997) Bias in meta-analysis detected by a simple, graphical test. BMJ 315:629–634. 10.1136/bmj.315.7109.6299310563 10.1136/bmj.315.7109.629PMC2127453

[35] Viechtbauer W, Cheung MWL (2010) Outlier and influence diagnostics for meta-analysisa. Res Synth Methods. 10.1002/jrsm.11. 1:112 – 2526061377 10.1002/jrsm.11

[36] Thompson SG, Higgins JP (2002) How should meta-regression analyses be undertaken and interpreted? Stat Med 21:1559–1573. 10.1002/sim.118712111920 10.1002/sim.1187

[37] Arczewska-Wlosek A, Swiatkiewicz S, Ognik K, Jozefiak D (2022) Effects of a Dietary Multi-Strain Probiotic and Vaccination with a Live Anticoccidial Vaccine on Growth Performance and Haematological, Biochemical and Redox Status Indicators of Broiler Chickens. Animals 12:3489. 10.3390/ani1224348936552409 10.3390/ani12243489PMC9774198

[38] Cai HM, Liao SQ, Li J, Liu QH, Luo SJ, Lv MN, Lin XH, Hu JJ, Zhang JF, Qi NS, Sun MF (2022) Single and combined effects of clostridium butyricum and coccidiosis vaccine on growth performance and the intestinal microbiome of broiler chickens. Front Microbiol 13. 10.3389/fmicb.2022.81142810.3389/fmicb.2022.811428PMC908312235547128

[39] Wang YY, Xu YB, Cao GT, Zhou XH, Wang Q, Fu AK, Zhan XA (2023) Bacillus subtilis DSM29784 attenuates Clostridium perfringens-induced intestinal damage of broilers by modulating intestinal microbiota and the metabolome. Front Microbiol 14:1138903. 10.3389/fmicb.2023.113890337007491 10.3389/fmicb.2023.1138903PMC10060821

[40] Ciszewski A, Jarosz LS, Kalinowski M, Marek A, Gradzki Z, Grabowski S, Hejdysz M, Nowaczewski S, Rysiak A (2022) Influence of Effective Microorganisms and Clinoptilolite on Gut Barrier Function, Intestinal Health and Performance of Broiler Chickens during Induced Eimeria tenella Infection. Agriculture-Basel 12:2176. 10.3390/agriculture12122176

[41] Cheng YH, Horng YB, Chen WJ, Hua KF, Dybus A, Yu YH (2021) Effect of Fermented Products Produced by Bacillus licheniformis on the Growth Performance and Cecal Microbial Community of Broilers under Coccidial Challenge. Animals 11:1245. 10.3390/ani1105124533925950 10.3390/ani11051245PMC8146065

[42] Yu YH, Wu CM, Chen WJ, Hua KF, Liu JR, Cheng YH (2021) Effectiveness of Bacillus licheniformis-Fermented Products and Their Derived Antimicrobial Lipopeptides in Controlling Coccidiosis in Broilers. Animals 11:3576. 10.3390/ani1112357634944351 10.3390/ani11123576PMC8698030

[43] Zhang TX, Qu HF, Zheng W, Zhang YA, Li YN, Pan TX, Li JY, Yang WT, Cao X, Jiang YL, Wang JZ, Zeng Y, Shi CW, Huang HB, Wang CF, Yang GL, Zhang JW, Wang N (2024) Oral vaccination with a recombinant Lactobacillus plantarum expressing the Eimeria tenella rhoptry neck 2 protein elicits protective immunity in broiler chickens infected with Eimeria tenella. Parasites Vectors 17:277. 10.1186/s13071-024-06355-w38943202 10.1186/s13071-024-06355-wPMC11212160

[44] Zhi WJ, Chen H, Bai BR, Jia ZP, Pan XH, Wang B, Kong R, Liu QJ, Ma CL, Ma DX (2022) Combined oral immunization with probiotics Entercoccus faecalis delivering surface-anchored Eimeria tenella proteins provide protective efficacies against homologous infection in chickens. Front Immunol 13:1042143. 10.3389/fimmu.2022.104214336311704 10.3389/fimmu.2022.1042143PMC9606674

[45] Barbalho R, Castaneda C, Araújo LF, Kiess AS, Carvalho RSB, Barbalho CB, Borges LL, Bonato MA (2023) Β-glucans and MOS, essential oil, and probiotics in diets of broilers challenged with Eimeria spp. and Clostridium perfringens. Poult Sci 102:102541. 10.1016/j.psj.2023.10254136893616 10.1016/j.psj.2023.102541PMC10011820

[46] Huang N, Ma Y, Chai J, Li Z, You X, Wang X, Huang Y, Shi H (2024) In ovo injection dosage of Lactobacillus rhamnosus on intestinal health and microbial composition of yellow broilers with or without Eimeria challenge. J Appl Poult Res 33:100411. 10.1016/j.japr.2024.100411

[47] Memon FU, Yang YQ, Zhang GY, Leghari IH, Lv FF, Wang YH, Laghari F, Khushk FA, Si HB (2022) Chicken Gut Microbiota Responses to Dietary Bacillus subtilis Probiotic in the Presence and Absence of Eimeria Infection. Microorganisms 10:1548. 10.3390/microorganisms1008154836013966 10.3390/microorganisms10081548PMC9412415

[48] Yang XL, Pan XH, Jia ZP, Bai BR, Zhi WJ, Chen H, Ma CL, Ma DX (2022) Oral administration of Lactobacillus brevis 23017 combined with ellagic acid attenuates intestinal inflammatory injury caused by Eimeria infection by activating the Nrf2/HO-1 antioxidant pathway. Vet Res 53:21. 10.1186/s13567-022-01042-z35303923 10.1186/s13567-022-01042-zPMC8931975

[49] Rodrigues RA, Silva LAM, Brugnera HC, Pereira N, Casagrande MF, Makino LC, Bragança CRS, Schocken-Iturrino RP, Cardozo MV (2024) Association of Bacillus subtilis and Bacillus amyloliquefaciens: minimizes the adverse effects of necrotic enteritis in the gastrointestinal tract and improves zootechnical performance in broiler chickens. Poult Sci 103:103394. 10.1016/j.psj.2023.10339438194830 10.1016/j.psj.2023.103394PMC10792630

[50] Cai HM, Luo SJ, Liu QH, Zhou QF, Yan ZQ, Kang Z, Liao SQ, Li J, Lv MN, Lin XH, Hu JJ, Yu SL, Zhang JF, Qi NS, Sun MF (2023) Effects of a complex probiotic preparation, Fengqiang Shengtai and coccidiosis vaccine on the performance and intestinal microbiota of broilers challenged with Eimeria spp. Parasites Vectors 16. 10.1186/s13071-023-05855-510.1186/s13071-023-05855-5PMC1037573937501177

[51] Jia L, Zhang X, Li X, Schilling MW, Peebles ED, Kiess AS, Zhang L (2022) Internal organ and skeletal muscle development in commercial broilers with woody breast myopathy. Poult Sci 101:102012. 10.1016/j.psj.2022.10201235896053 10.1016/j.psj.2022.102012PMC9326126

[52] Jia L, Zhang X, Li X, Schilling W, David Peebles E, Kiess AS, Zhai W, Zhang L (2022) Bacitracin, Bacillus subtilis, and Eimeria spp. challenge exacerbates woody breast incidence and severity in broilers. Poult Sci 101:101512. 10.1016/j.psj.2021.10151234788711 10.1016/j.psj.2021.101512PMC8605194

[53] Poudel S, Zhang L, Tabler GT, Lin J, Zhai W (2021) Effects of riboflavin and Bacillus subtilis on internal organ development and intestinal health of Ross 708 male broilers with or without coccidial challenge. Poult Sci 100:100973. 10.1016/j.psj.2020.12.07033588345 10.1016/j.psj.2020.12.070PMC7896149

[54] Yang Y, Memon FU, Hao K, Jiang M, Guo L, Liu T, Lv F, Zhang W, Zhang Y, Si H (2021) The combined use of Bacillus subtilis-based probiotic and anticoccidial herb had a better anti-Eimeria tenella efficiency. J Appl Poult Res 30:100181. 10.1016/j.japr.2021.100181

[55] Yulianto AB, Suwanti LT, Widiyatno TV, Suwarno S, Yunus M, Tyasningsih W, Hidanah S, Sjofjan O, Lokapirnasari WP (2021) Probiotic Pediococcus pentosaceus ABY 118 to Modulation of ChIFN- γ and ChIL-10 in Broilers Infected by Eimeria tenella Oocyst. Vet Med Int 2021:1473208. 10.1155/2021/147320834659734 10.1155/2021/1473208PMC8519706

[56] Aida M, Yamada R, Nakamura S, Imaoka T, Shimonishi H, Matsuo T, Taniguchi I, Tsukahara T (2022) The Effect of Supplementation with Weizmannia coagulans Strain SANK70258 to Coccidia-Infected Broilers Is Similar to That of a Coccidiostat Administration. Vet Sci 9:406. 10.3390/vetsci908040636006321 10.3390/vetsci9080406PMC9416079

[57] Chalalai T, Promsut W, Hinkhao K, Hengphrathani T, Sangsakul K, Bhavabhutanon N, Nonkookhetkhong T (2025) Effects of probiotics and amprolium on performance, lesion scores, oocyst shedding, and histopathological changes in Eimeria tenella-infected broiler chickens. Vet World 18:1400–1410. 10.14202/vetworld.2025.1400-141040689190 10.14202/vetworld.2025.1400-1410PMC12269941

[58] Chen P, Lv H, Liu W, Wang Y, Zhang K, Che C, Zhao J, Liu H (2023) Effects of Lactobacillus plantarum HW1 on Growth Performance, Intestinal Immune Response, Barrier Function, and Cecal Microflora of Broilers with Necrotic Enteritis. Animals 13:3810. 10.3390/ani1324381038136847 10.3390/ani13243810PMC10740588

[59] de Souza OF, Vecchi B, Gumina E, Matté F, Gazoni FL, Hernandez-Velasco X, Hall JW, Stefanello C, Layton S (2022) Development and Evaluation of a Commercial Direct-Fed Microbial (Zymospore^®^) on the Fecal Microbiome and Growth Performance of Broiler Chickens under Experimental Challenge Conditions. Animals 12:1436. 10.3390/ani1211143635681899 10.3390/ani12111436PMC9179881

[60] Li P, Zheng L, Qi Y, Liu Z, Du E, Wei J, Zhang Z, Guo S, Ding B (2022) Dietary Lactobacillus fermentum and Lactobacillus paracasei improve the intestinal health of broilers challenged with coccidia and Clostridium perfringens. Front Vet Sci 9:1025677. 10.3389/fvets.2022.102567736590818 10.3389/fvets.2022.1025677PMC9797813

[61] Nooreh Z, Taherpour K, Ghasemi HA, Gharaei MA, Shirzadi H (2021) Protective and immunostimulatory effects of in-feed preparations of an anticoccidial, a probiotic, a vitamin-selenium complex, and Ferulago angulata extract in broiler chickens infected with Eimeria species. BMC Vet Res 17:307. 10.1186/s12917-021-03005-634526018 10.1186/s12917-021-03005-6PMC8442408

[62] Ogwiji M, Jatau ID, Natala AJ, Mohany M, Al-Rejaie SS, Zhu M (2024) Effect of prebiotic, probiotic, and synbiotic products on oxidative status, performance, and parasitological parameters of broiler chickens induced with cecal coccidiosis. J Appl Poult Res 33:100472. 10.1016/j.japr.2024.100472

[63] Osho S, Bolek K, Saddoris-Clemons K, Humphrey B, Garcia M (2023) Impact of a direct-fed microbial supplementation on intestinal permeability and immune response in broiler chickens during a coccidia challenge. Front Microbiol 14. 10.3389/fmicb.2023.128339310.3389/fmicb.2023.1283393PMC1064401038029093

[64] Poudel S, Tabler GT, Lin J, Zhai W, Zhang L (2022) Riboflavin and Bacillus subtilis effects on growth performance and woody-breast of Ross 708 broilers with or without Eimeria spp. challenge. J Anim Sci Technol 64:443–461. 10.5187/jast.2022.e2435709099 10.5187/jast.2022.e24PMC9184709

[65] Sun L, Liu Y, Xiao P, Zhang K, Bai S, Wang J, Zeng Q, Peng H, Mu Y, Xuan Y (2024) Probiotic Bacillus subtilis QST713 improved growth performance and enhanced the intestinal health of yellow-feather broilers challenged with coccidia and Clostridium perfringens. Poult Sci 103:104319. 10.1016/j.psj.2024.10431939353329 10.1016/j.psj.2024.104319PMC11472712

[66] Van der Klein S, Arora S, Haldar S, Dhara A, Gibbs K (2024) A dual strain probiotic administered via the waterline beneficially modulates the ileal and cecal microbiome, sIgA and acute phase protein levels, and growth performance of broilers during a dysbacteriosis challenge. Poult Sci 103:104462. 10.1016/j.psj.2024.10446239504831 10.1016/j.psj.2024.104462PMC11577228

[67] Wang Y, Lv XG, Li XM, Zhao JS, Zhang K, Hao XJ, Liu KD, Liu HW (2021) Protective Effect of Lactobacillus plantarum P8 on Growth Performance, Intestinal Health, and Microbiota in Eimeria-Infected Broilers. Front Microbiol 12:705758. 10.3389/fmicb.2021.70575834305875 10.3389/fmicb.2021.705758PMC8299996

[68] Wang YY, Xu YB, Xu SL, Yang JY, Wang KY, Zhan XA (2021) Bacillus subtilis dsm29784 alleviates negative effects on growth performance in broilers by improving the intestinal health under necrotic enteritis challenge. Front Microbiol 12. 10.3389/fmicb.2021.72318710.3389/fmicb.2021.723187PMC848178234603247

[69] Zhang RT, Yang J, Wang QJ, Hu DD, Zhao QP, Zhu SH, Qiao Y, Zhao FH, Wang ZC, Wang JW, Yu Y, Han HY, Hao LL, Dong H (2024) Comparative Efficacy of Plant Extracts and Probiotics on Growth and Gut Health in Chickens with Necrotic Enteritis. Animals 14:3312. 10.3390/ani1422331239595364 10.3390/ani14223312PMC11591468

[70] van der Klein SAS, Bernardeau M, Wang Q, Gibbs K (2025) A cross-study analysis of the effect of a dual-strain probiotic applied via the waterline on the growth performance and gut health of broilers under a mild necrotic enteritis challenge. Poult Sci 104:104550. 10.1016/j.psj.2024.10455039626605 10.1016/j.psj.2024.104550PMC11647604

[71] Arczewska-Włosek A, Świątkiewicz S, Ognik K, Józefiak D (2022) Effects of a dietary multi-strain probiotic and vaccination with a live anticoccidial vaccine on growth performance and haematological, biochemical and redox status indicators of broiler chickens. Animals 12:348936552409 10.3390/ani12243489PMC9774198

[72] Mohsin M, Zhang Z, Yin G (2022) Effect of probiotics on the performance and intestinal health of broiler chickens infected with Eimeria tenella. Vaccines 10:97. 10.3390/vaccines1001009735062758 10.3390/vaccines10010097PMC8778926

[73] Wang Y, Lv X, Li X, Zhao J, Zhang K, Hao X, Liu K, Liu H (2021) Protective effect of Lactobacillus plantarum P8 on growth performance, intestinal health, and microbiota in Eimeria-infected broilers. Front Microbiol 12:705758. 10.3389/fmicb.2021.70575834305875 10.3389/fmicb.2021.705758PMC8299996

[74] Shah BR, Al Hakeem WG, Shanmugasundaram R, Selvaraj RK (2025) A comparative evaluation of antibiotic and synbiotic supplementation on production performance and necrotic enteritis severity in broilers during an experimental necrotic enteritis challenge. Front Physiol 15:1511380. 10.3389/fphys.2024.151138039882325 10.3389/fphys.2024.1511380PMC11774996

[75] Ogbuewu IP, Mabelebele M, Sebola NA, Mbajiorgu C (2022) Bacillus probiotics as alternatives to in-feed antibiotics and its influence on growth, serum chemistry, antioxidant status, intestinal histomorphology, and lesion scores in disease-challenged broiler chickens. Front Vet Sci 9:876725. 10.3389/fvets.2022.87672535573393 10.3389/fvets.2022.876725PMC9096611

